# Inhibition of NEDD8 NEDDylation induced apoptosis in acute myeloid leukemia cells via p53 signaling pathway

**DOI:** 10.1042/BSR20220994

**Published:** 2022-08-10

**Authors:** Yanli Chen, Ling Sun

**Affiliations:** Department of Hematology, the First Affiliated Hospital of Zhengzhou University, Zhengzhou, Henan 450052, P.R. China

**Keywords:** AML, MLN4924, NEDD8, p21, p53

## Abstract

MLN4924 is a potent and selective small-molecule inhibitor of NEDD8-activating enzyme, which showed antitumor effect in several types of malignant tumor types. However, the mechanism of action of MLN4924 in acute myeloid leukemia (AML) requires further investigation. Real-time fluorescent quantitative polymerase chain reaction (RT-qPCR) was conducted to detect the mRNA levels of genes. Gene expression was knocked down by short hairpin RNA (shRNA). Moreover, the protein expression was detected by Western blotting (WB) assay. The proliferation and apoptosis of AML cells were measured by Cell Counting Kit-8 (CCK8) assay and flow cytometry (FCM). In the present study, we observed that the mRNA expression levels of *NEDD8*, *UBA3*, *UBE2M* and *RBX1* in AML patients were up-regulated compared with healthy controls, which were correlated with worse overall survival (OS) of patients. Besides, knockdown of *UBA3*, *UBE2M* and *RBX1* inhibited the NEDDylation of CULs and increased the protein expression of p53 and p21 in MOLM-13 cell line. In AML cells, MLN4924 inhibited cell proliferation, promoted cell apoptosis, and induced cell cycle arrest at the G_2_/M phase. As revealed by experiments *in vivo* and *in vitro*, the NEDDylation of CULs was significantly inhibited and the p53 signaling pathway was activated after MLN4924 treatment. So, we concluded that *NEDD8*, *UBA3*, *UBE2M* and *RBX1* may serve as the prognostic biomarkers and novel therapeutic targets for AML. Inhibition of the NEDDylation pathway resulted in an anti-leukemia effect by activating the p53 signaling pathway.

## Introduction

NEDD8 (neural precursor cell-expressed developmentally down-regulated 8) is a small ubiquitin-like molecule, which can covalently regulate the activity of CRLs (cullin-ring ligases, the largest family of E3 ubiquitin ligases). NEDD8 is similar to ubiquitin in structure, which shares 60% identity and 80% homology to ubiquitin and is encoded by 81 amino acids [1]. First of all, NEDD8 is synthesized as a precursor molecule with 5 residues in the downstream of glycine at position 76 (Gly76); thereafter, it is lysed into a mature NEDD8 molecule by a C-terminal hydrolase [1]. Mature NEDD8 molecules can be activated by the E1 (NEDD8-activating enzyme (NAE), which contains the UBA3/UBE1C and APPBP1/NAE1 subunits). Afterwards, a high-energy thioester bond is formed between the C-terminal Gly76 of NEDD8 and the UBA3 cysteine active site. The activated NEDD8 is later transferred to the cysteine active site of a NEDD8 conjugation enzyme E2 (UBE2M/UBC12 or UBE2F/NCE2) for the formation of a thioester bond [2,3]. Finally, by the involvement of E3 ligase, the glycine at position 76 of the NEDD8 C-terminal binds to the lysine residue of the substrate via an isopeptide bond. The post-translational modification of the protein bound to NEDD8 is called NEDDylation, which is an important biochemical process employed to regulate protein function. Notably, the most common substrate for NEDDylation is the CUL subunit of CRLs, which covalently binds NEDD8 to CUL as well as other target molecules via a cascade reaction that involves the E1 activating enzyme, the E2 conjugation enzyme and the E3 ligase [4,5]. Some CRL substrates, such as signal transducers, cell cycle regulators, transcription factors, tumor suppressors, and oncoproteins, are the key regulatory molecules with short lifespans [6–9]. CRLs regulate numerous biological processes by targeting degradation [5–7]. There are approximately ten types of NEDD8 E3 ligases, most of which possess a RING domain. Thus, CUL NEDDylation is a process that activates CRLs to promote the ubiquitination of their protein substrates.

MLN4924 is a newly discovered inhibitor of NAE [8]. It forms a covalent adduct with NEDD8, which is catalyzed by NAE. The MLN4924-NEDD8 adduct is similar to the adenosylated NEDD8 in that it closely binds to the active site of NAE and blocks the activity of NAE, thus preventing subsequent enzymatic reactions [10,11]. Only one NAE can catalyze the first step of NEDDylation, and its inhibitor MLN4924 should block the entire NEDDylation pathway. As a matter of fact, MLN4924 effectively inhibits the NEDDylation of CULs [12,13], including CUL1-CUL3, CUL4A, CUL4B and CUL5 [10]. Given that CUL NEDDylation is required for the activities of CRLs, which are abnormally activated in human cancers [4,14], MLN4924 inactivates the entire family of CRL E3 ligases by blocking the NEDDylation of CULs [5]. Consequently, several key CRL substrates accumulate, which trigger a variety of cellular responses leading to cell cycle arrest, apoptosis, senescence and autophagy in a cell type-dependent manner.

The NEDDylation pathway was excessively activated in many human tumor tissues compared with adjacent normal tissues [15–22], and the overexpression of enzymes associated with NEDD8 NEDDylation was associated with disease progression [16–18,20,21]. Studies had shown that inhibition of NEDD8 pathway had a very good antitumor effect [23–29]. Currently, chemotherapy is still the main treatment for acute myeloid leukemia (AML). AML patients have a high rate of chemotherapy resistance and relapse, and a low remission rate after relapse. The median overall survival (mOS) of relapsed or refractory AML (R/R AML) patients from relapse was about 6 months and the 5-year OS was only 10% [30]. Treatment for R/R AML remains challenging [31]. Members of our team conducted some studies on NEDD8 NEDDylation in solid tumors and found that MLN4924 exhibited a good inhibitory effect on solid tumors. In this context, we wanted to observe the effect of MLN4924 on AML and explore its mechanism. The present study aimed to explore the activation of molecules associated with the NEDDylation pathway in AML and examine the effect of inhibiting NEDDylation on AML cells.

## Materials and methods

### Collection of bone marrow samples

A total of 128 samples of bone marrow were collected from AML patients from September 2018 to September 2019 in the First Affiliated Hospital of Zhengzhou University (China). The samples included 109 cases of initially diagnosed AML and 19 cases of refractory-recurrent AML. There were 73 men and 55 women in the cohort, with the age of 14–86 (median, 47.5) years. In addition, 16 normal bone marrow samples were collected from healthy subjects in the outpatient clinics, including 9 men and 7 women, with the age of 24–72 (median, 38.5) years. The clinical characteristics of both patient and control groups were shown in Table 1. All participants provided the written informed consents. Ethics approval was obtained from the Human Research Ethics Committee of the First Affiliated Hospital of Zhengzhou University.

**Table 1:** Basic clinical characteristics of all participants

Characteristics	AML group	Control group
Age/years, median (range)	47.5 (14–86)	38.5 (24–72)
Age group/*n* (%)		
≥60 years	30 (23.4)	6 (37.5)
<60 years	98 (76.6)	10 (62.5)
Gender/*n* (%)		
Men	73 (57.8)	9 (56,2)
Women	55 (42.2)	7 (43.8)
FAB subtypes/*n* (%)		
M_0_	4 (3.1)	
M_1_	6 (4.7)	
M_2_	68 (53.1)	
M_4_	15 (11.7)	
M_5_	33 (25.8)	
M_6_	1 (0.8)	
M_7_	1(0.8)	
Prognosis Risk/*n* (%)		
Good	20 (15.7)	
Intermediate	59(46)	
Poor	49 (38.3)	
Cytogenetics/*n* (%)		
Normal	63 (49.2)	
Complex	10 (7.8)	
inv(16) or t(16;16)/CBFβ/MYH11	5 (3.9)	
t(8;21)/AML1/ETO	21 (16.4)	
11q23/MLL	8 (6.2)	
t(9;22)/BCR/ABL1	3 (2.4)	
Others	18 (14.1)	
Molecular genetics/*n* (%)		
FLT3-ITD	23 (17.9)	
c-Kit	14 (10.9)	
NPM-1	20 (15.6)	
TP53	2 (1.5)	
RUNX1	3 (2.3)	
ASXL1	7 (5.5)	
DNMT3a	7 (5.5)	
SF3B1	1 (0.8)	
CEBPA double mutation	7 (5.5)	
IDH1	8 (6.3)	
IDH2	8 (6.3)	

### Reverse transcription-quantitative PCR (RT-qPCR)

Mononuclear cells were separated by the use of lymphocyte separation medium (HaoYang, China). Thereafter, total RNA was extracted from mononuclear cells using TRIzol® (Thermo Fisher Scientific, Inc.). Later, the genomic DNA was removed from RNase-free RNA I (Thermo Fisher Scientific, Inc), and 1 μg RNA was reverse-transcribed into cDNA with the RevertAid First Strand cDNA Synthesis kit (Thermo Fisher Scientific, Inc.). Next, RT-qPCR (Maxima SYBR Green qPCR Master Mix; Thermo Fisher Scientific, Inc.) was carried out to detect the expression of molecules related to the NEDDylation pathway. The primers were synthesized by Sangon Biotech (Shanghai) Co. Ltd. The primer sequences were listed below.

For NEDD8: forward, 5′-CAGAGGCTCATCTACAGTGGCA-3′; reverse, 5′-GTCCATCACTGCCTAAGACCAC-3′. For UBA3: forward, 5′-AATCTCCAGCCATCACAGCCAC-3′; reverse, 5′-GTGACATCAGCAACCGCCAGTT-3′. For UBE2M: forward, 5′-AGCCAGTCCTTACGATAAACTCC-3′; reverse, 5′-TGCACGTTCTGCTCAAACAGCC-3′. For RBX1: forward, 5′-ACTGTGCCATCTGCAGGAACCA-3′; reverse, 5′- ACCTGTCGTGTTTTGAGCCAGC-3′. For β-actin: forward, 5′-ATCATGTTTGAGACCTTCAACA-3′; reverse, 5′-CATCTCTTGCTCGAAGTCCA-3′.

The reaction system consisted of 5 μl diluted cDNA, 12.5 μl Maxima SYBR Green qPCR Master Mix (2×), 0.3 μM Forward and Reverse primers, and 10 nM ROX Solution. Finally, the reaction mixture was diluted with nuclease-free H_2_O to a final volume of 25 μl. The cycling conditions were shown below, 10 min of initial denaturation at 95°C, 15 s of denaturation at 95°C, 30 s of annealing at 60°C, and 30 s of extention at 72°C for a total of 41 cycles. The Quant Studio 5 real-time PCR system was used for RT-qPCR (Thermo Fisher Scientific, Inc.), with β-actin being the control gene. The *C*q values were obtained from the RT-qPCR results, and the relative expression level of each gene was calculated using the 2^−ΔΔCq^ method.

### Cell lines and cell culture

THP-1 cells were purchased from Xiehe Cell Bank of Beijing, MOLM-13 cells were presented by the Second Affiliated Hospital of Zhejiang University, and 293 T cells were presented by the Cell Therapy Center of the First Affiliated Hospital of Zhengzhou University. In addition, the fresh bone marrow samples were collected from two patients with newly diagnosed AML, and mononuclear cells were isolated with the lymphocyte separation medium. These were used as the primary AML cell samples. Afterwards, all the AML cells were cultured in RPMI-1640 medium (MilliporeSigma), whereas 293 T cells were cultured in DMEM both of which contained 10% fetal bovine serum (FBS), 1% penicillin and streptomycin (Gibco; Thermo Fisher Scientific, Inc.). The cells were cultured at a constant temperature of 37°C and 5% CO_2_, and fresh medium was replaced every three days. Cells at logarithmic growth phase were harvested for subsequent experiments.

### Preparation of the lentivirus and shRNA-mediated knockdown

The short hairpin RNA (shRNA) vectors (GV248-GFP) targeting respectively *UBA3*, *UBE2M* and *RBX1* respectively were constructed by Shanghai GeneChem Co., Ltd. Typically, three shRNA vectors were designed for each gene. Meanwhile, an empty plasmid served as the negative control. The RNAi target sequences were listed below.

UBA3-RNAi#1(34824-1): ccTCTATTGAAGAACGAACAA; UBA3-RNAi#2(34825-1): gcCTGGAATGACTGCTTGTAT; UBA3-RNAi#3(34826-1): cgACACTTTCTATCGACAATT; UBE2M-RNAi#1(80842-1): CGGCTGTTTGAGCAGAACGTG; UBE2M-RNAi#2(80843-1): TACATCGGCTCCACCTACTTT; UBE2M-RNAi#3(80844-1): GAGGTCCCACCAGGCTATTAA; RBX1-RNAi#1(41656-11): ctGCATCTCTCGCTGGCTCAA; RBX1-RNAi#2(41658-1): atGTCAAGCTAACCAGGCGTC; and RBX1-RNAi#3(41659-1): ctTTCCCTGCTGTTACCTAAT.

The second generation lentivirus packaging system was used. In brief, the shRNA vectors (25 μg) were mixed with the packaging vectors pSPAX2 (8 μg) and pMD2.G (4 μg) (presented by the Cell Therapy Center of the First Affiliated Hospital of Zhengzhou University). Thereafter, Lipofectamine® 3000 Transfection reagent (Thermo Fisher Scientific, Inc.) was added into the above mixture, and the sample was added dropwise into the dish (diameter: 10 cm) containing 10 ml DMEM, where the 293T cells were in adherent growth. After 72 h, the viral supernatant was harvested by ultrarcentrifugation in the Beckman centrifuge at 4°C and 25,000 rpm for 4 h. The lentiviruses were later transfected into MOLM-13 cells with polybrene by centrifugation at 1500 ***g*** and room temperature for 90 min in a sterile environment. Thereafter, the puromycin was added to screen the MOLM-13 cells. The gene knockdown efficiency was assessed by RT-qPCR. Then, the shRNA vectors with the highest inhibitory rate were selected for subsequent experiments according to the RT-qPCR results. Besides, the protein expression of target gene was detected by Western blotting (WB) assay, and the effect of gene knockdown on the NEDDylation of CULs, p53 and p21 was also determined.

### Detection of cell proliferation, apoptosis and cell cycle

THP-1 and MOLM-13 cells, as well as the primary AML cells were treated with MLN4924 (MedChemExpress) for 24, 48 and 72 h, respectively. The 0.01% dimethyl sulfoxide (DMSO) was used as a negative control. The effect of MLN4924 on cell proliferation was detected by Cell Counting Kit-8 (CCK-8, Higashi, Japan) assay. The absorbance at 450 nm was measured after CCK-8 was added to AML cells for 4 h at a constant temperature of 37°C and 5% CO_2_. Triplicate wells were set up for each condition.

After THP-1 and MOLM-13 cells were treated with MLN4924 for 24 h, the cell apoptosis and cell cycle progression were analyzed using a FACSCanto™ II, flow cytometer (BD Biosciences). Additionally, Annexin V-FITC/PI Apoptosis Detection Kit and Cell Cycle Detection Kit (Nanjing KeyGen Biotech Co., Ltd.) were utilized to analyze the apoptosis and cell cycle progression, respectively. These assays were performed in triplicate. FlowJo 10.0.7 was employed for data analysis (FlowJo LLC).

### Protein extraction and WB assay

AML cells were lysed with the cell lysis buffer containing protease inhibitors (Roche, Diagnostics). Later, the proteins were extracted and the protein concentration was detected by dicarboxylic acid (BCA) assay (Beijing Solarbio Science & Technology Co., Ltd). The protein loading was 30 μg/20 μl. Thereafter, protiens were separated by sodium dodecyl sulfate polyacrylamide gel electrophoresis (SDS-PAGE) on the 10% separation gels (at 80 V for 100 min), then transferred on to the polyvinylidene fluoride (PVDF) membranes (MilliporeSigma) at 200 mA for 2 h. Then the PVDF membranes were incubated in 5% skim milk powder at room temperature for 2 h. After overnight incubation with primary antibodies (Abcam) at 4°C, the memebranes were incubated with secondary antibodies (Abcam) at room temperature for 1 h. The primary antibodies used in the present study were as follows. Anti-Cullin 1 (product code ab75817; dilution 1:1,000; Abcam), anti-Cullin 2 (product code ab166917; dilution 1:1,000; Abcam), anti-Cullin 3 (product code ab75851; dilution 1:20,000; Abcam), anti-Cullin-4A (product code ab92554; dilution 1:20,000; Abcam), anti-Cullin-4B (product code ab227724; dilution 1:500; Abcam), anti-Cullin 5 (product code ab184177; dilution 1:10,000, Abcam), anti-NEDD8 (product code ab81264; dilution 1:1,000, Abcam), anti-UBA3 (product code ab38649; dilution 1:500; Abcam), anti-UBE2M (product code ab109507; dilution 1:10,000; Abcam), anti-RBX1 (product code ab133565; dilution 1:1000; Abcam), anti- GAPDH (product code ab8245; dilution 1:5000; Abcam), anti-p53 (product no. 10442-1-AP; dilution 1:1000; proteintcch, China) and anti-p21 (product no. 10355-1-AP; dilution 1:1000; proteintcch, China). The secondary antibodies used included anti-Mouse IgG (product code ab6728; dilution 1:5,000; Abcam) and anti-Rabbit IgG (product code ab6721; dilution 1:5,000; Abcam). The protein bands were detected using the enhanced chemiluminescence (ECL) reagent (Affinity Company). The AI600 control software (version 1.2.0; Cytiva) was utilized to analyze the staining intensity.

### Establishment of tumor xenograft mouse model

The 4- to 5-week-old specific-pathogen-free (SPF) female NOD/SCID mice weighing 16.5 ± 2 g were purchased from Beijing Weitonglihua Laboratory Animal Technology Co., Ltd. All mice met the requirements of quality inspection and were kept in the SPF animal house. The health and behaviors of mice were monitored once a week by Henan Academy of Medical and Pharmacology Sciences (Zhengzhou University). Thereafter, the mice were randomly divided into two groups, namely, control group (*n*=5) and experimental group (*n*=5). MOLM-13 cells were selected and marked with the luciferase gene by lentivirus packaging and transfection. Next, 1 × 10^6^ MOLM-13 cells (0.1 ml) were injected into the right flank of NOD/SCID mice to establish the AML xenograft tumor model. The xenograft tumor was visible after 1 week. Mice were given intraperitoneal injection of luciferin (Beijing Solarbio Science & Technology Co., Ltd) and anesthetized with 2.5% isoflurane inhalation in an anesthesia cage (Matrx Animal Anesthesia Ventilator System). After 15 min, the xenograft tumor was examined *in vivo* using an animal imaging system (PerkinElmer Inc.). On the following day, 60 mg/kg MLN4924 dissolved in corn oil (MedChemExpress) was intraperitoneally injected into each experimental mouse twice a day for 5 days a week for 2 weeks. At the same time, vehicle (corn oil) was used to treat control mice by the same methods. The size of the xenograft tumor was measured using a caliper and the small animal in imaging system *in vivo*. Fourteen days after MLN4924 injection when the maximum tumor diameter reached nearly 20 mm, all the mice were killed by intraperitoneal injection with 1% pentobarbital sodium (150 mg/kg) to achieve cardiac arrest. Thereafter, xenograft tumors were isolated from the mice and positioned in liquid nitrogen for quick freezing. Then, the total proteins were extracted from the xenograft tumors for WB analysis. The effect of MLN4924 on the expression of NEDD8-CULs, p21 and p53 in mice was detected by WB assay. All the animal experiments were performed in Henan Academy of Medical and Pharmacology Sciences and in accordance with the U.K. Animals (Scientific Procedures) Act (1986) and associated guidelines, as well as the EU Directive 2010/63/EU for animal experiments. The experiments were approved by the Institutional Animal Care and Use Committee of the First Affiliated Hospital of Zhengzhou University.

### Statistical analysis

The SPSS 23.0 software (IBM Corp.) was employed to analyze the experimental data. All data were represented by mean ± standard deviation (SD). The means between two groups were compared by an independent sample *t*-test, while those among multiple groups were compared by one-way ANONA and LSD-*t* test. Chi-square test was adopted to compare qualitative data. The Kaplan–Meier (KM) method was utilized to draw the survival curves of patients, and log-rank tests were used to compare the survival rates. Both univariate and multivariate Cox repression analyses were conducted to determine whether the expression of the interested genes was the independent factor for overall survival (OS). Statistical analysis was conducted at an inspection level of *α*=0.05, where *P*<0.05 was considered statistically significant, *P*<0.01, statistically significant difference and *P*<0.001, extremely significant statistical difference.

## Results

### *NEDD8, UBA3, UBE2M* and *RBX1* were over-expressed in AML patients and were correlated with worse OS

**Figure 1:**
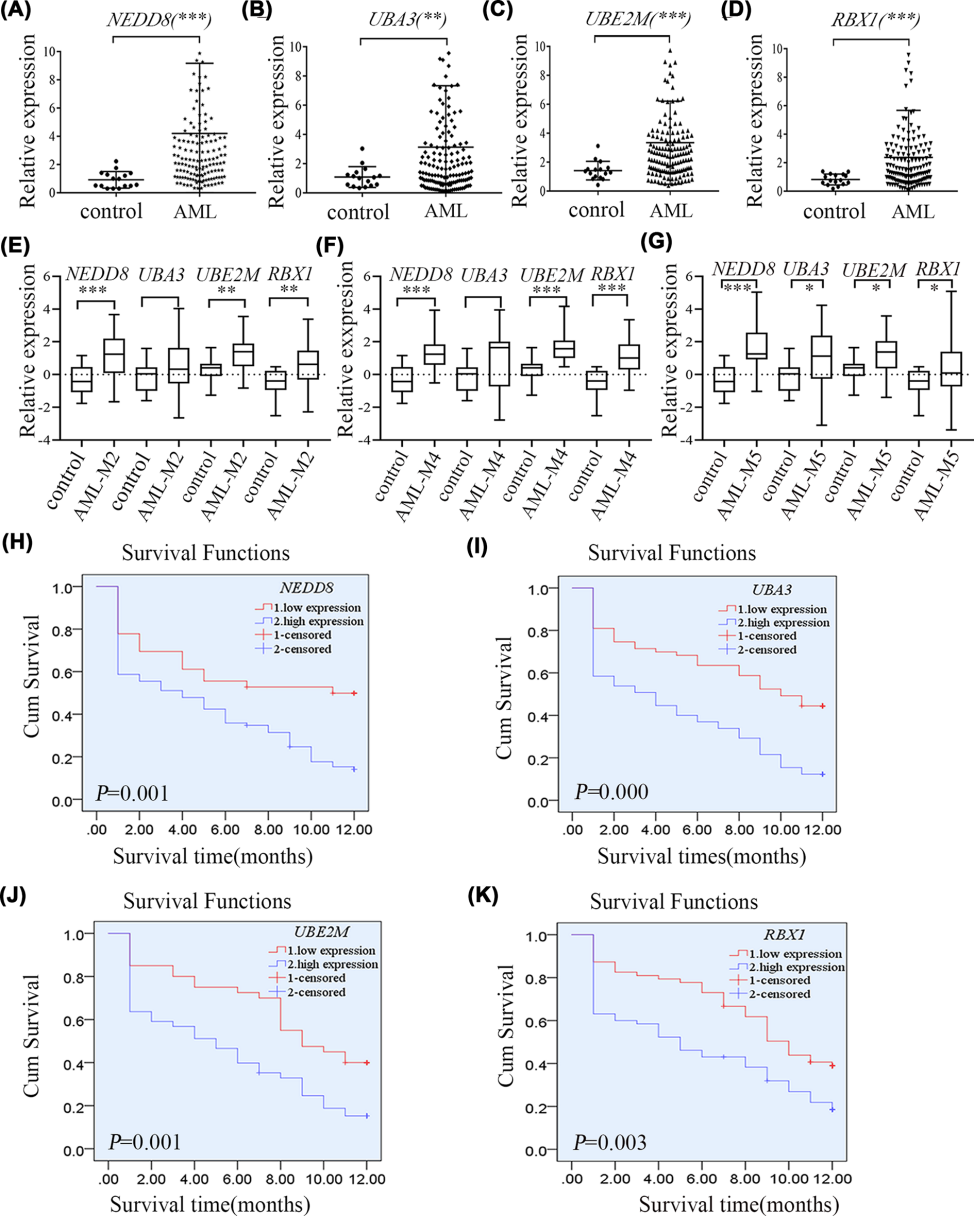
*NEDD8, UBA3, UBE2M* and *RBX1* were over-expressed in AML patients and were correlated with worse OS (**A–D**) Compared with normal control, the mRNA levels of *NEDD8*, *UBA3*, *UBE2M* and *RBX1* were significantly higher in AML patients. (**E–G**) As revealed by FAB subtype analysis, the mRNA expression levels of *NEDD8*, *UBE2M* and *RBX1* were higher in patients with AML-M2, M4 and M5 than those in healthy controls. In patients with AML-M5, the mRNA expression of UBA3 was higher than that in the control group. (**H–K**) Survival analysis revealed that the OS rate of patients with overexpression of *NEDD8*, *UBA3*, *UBE2M* and *RBX1* was lower than that of patients with low-expression. ROC curves were plotted based on the mRNA expression levels of *NEDD8*, *UBA3*, *UBE2M* and *RBX1*, respectively, and Jorden index was calculated to determine the cut-off value (*NEDD8*, 1.4257; *UBA3*, 1.6511; U*BE2M*, 1.6146; *RBX1*, 1.4026) to divide the samples into high and low expression groups. An independent sample *t*-test was conducted to compare the means between the two groups. Triplicates were set for each gene in each sample for RT-qPCR. Chi-square test was performed to compare the qualitative data. The KM method was employed to draw the survival curves of patients, and log-rank tests were utilized to compare the survival rates. Univariate and multivariate Cox analyses were adopted to determine whether the expression of the interested genes was the independent prognostic factor for OS; ***P<0.001, **P<0.01, *P<0.05.

To investigate the expression of genes associated with the NEDD8 pathway in AML, bone marrow samples were collected from 128 AML patients and 16 healthy controls. According to our results, there was no significant difference in gender distribution (*P*=0.953) or average age (*P*=0.493) between AML and control groups. Meanwhile, the expression of *NEDD8* mRNA was significantly higher in AML patients than in healthy controls (*P*<0.001, Figure 1A). In addition, *UBA3*, *UBE2M* and *RBX1* mRNA expression significantly increased in AML patients (*P*<0.01, Figure 1B–D). As revealed by French–American–British (FAB) subtype analysis, the mRNA expression levels of *NEDD8*, *UBA3*, *UBE2M* and *RBX1* were higher in AML-M5 patients than in controls (*P*<0.05, Figure 1G). In patients with AML-M4 and AML-M2, the mRNA levels of *NEDD8*, *UBE2M* and *RBX1* were higher than those in controls (*P*<0.001); however, there was no significant difference in the UBA3 mRNA expression (*P*>0.05, Figure 1E,F). Statistical analysis was not performed in the other FAB subtypes because of the small sample size. In patients carrying the *AML1/ETO* fusion gene, the *UBA3* mRNA expression levels were up-regulated compared with controls (*P*<0.05), while those of *NEDD8*, *UBE2M* and *RBX1* did not show any significant difference (*P>*0.05). No statistical analysis was performed due to the small number of patients carrying the *CBFβ/MYH11* fusion gene.

Moreover, survival analysis was performed to further investigate the roles of these up-regulated genes. Receiver operating characteristic (ROC) curves were plotted based on the mRNA expression levels of *NEDD8*, *UBA3*, *UBE2M* and *RBX1*, respectively. In addition, Jorden index was calculated to determine the cut-off value (*NEDD8*, 1.4257; *UBA3*, 1.6511; *UBE2M*, 1.6146; *RBX1*, 1.4026), which was later used as the criterion to divide samples into the high- and low-expression groups. KM analysis showed that the OS rate of AML patients with high *NEDD8* expression was lower than that of patients with low *NEDD8* expression (*P*<0.01, log-rank test, Figure 1H). Moreover, the results of univariate and multivariate Cox regression analyses showed that over-expression of *NEDD8* was an independent prognostic factor for OS in AML (*P*<0.01). Age and sex were not identified as the independent risk factors affecting the OS of AML patients with over-expression of *NEDD8* (*P*>0.05). Similarly, over-expression of *UBA3*, *UBE2M* and *RBX1* was associated with worse survival of AML patients (*P*<0.001, log-rank test, Figure 1I–K), which was an independent prognostic factor for OS in AML (*P*<0.01).

### *UBA3, UBE2M* and *RBX1* knockdown inhibited the NEDD8 NEDDylation of CULs and activated the p53 signaling pathway in MOLM-13 cells

**Figure 2:**
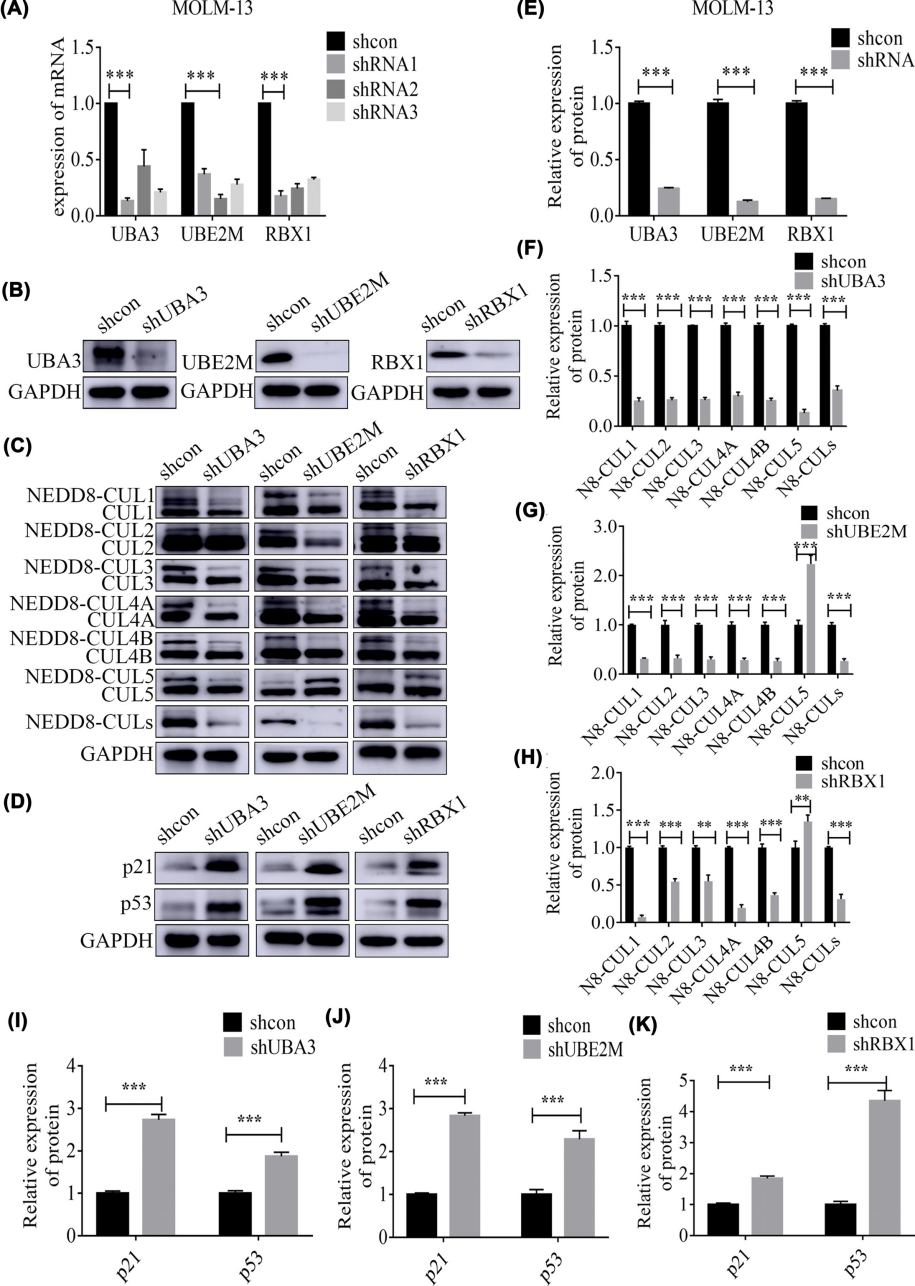
*UBA3*, *UBE2M* and *RBX1* knockdown inhibited the NEDD8 NEDDylation of CULs and activated the p53 signaling pathway in MOLM-13 cells (**A**) The knockdown efficiency of shRNA was >70% by RT-qPCR. (**B** and **E**) After transfection of shRNA in MOLM-13 cells, the expression of UBA3, UBE2M and RBX1 proteins was reduced. (**C**) and (**F–H**) NEDD8 NEDDylation of CUL1-4 was significantly inhibited following the knockdown of UBA3, UBE2M and RBX1. The NEDDylation of CUL5 was suppressed by the knockdown of *UBA3* but up-regulated by the knockdown of *UBE2M* and *RBX1*. (**D**) and (**I–K**) The expression of p21 and p53 significantly increased following UBA3, UBE2M and RBX1; ***P<0.001, **P<0.01. An independent sample *t-*test was adopted to compare the means between two groups. Each experiment was carried out in triplicate.

To explore the functions of UBA3, UBE2M and RBX1, these three targets were knocked down by shRNA lentiviral transfection. The knockdown efficiency was >70%, as evidenced by RT-qPCR (Figure 2A, *P*<0.001). Thereafter, the shRNA plasmid with the highest inhibitory rate was selected for subsequent experiments. After transfection, the expression of UBA3, UBE2M and RBX1 proteins was significantly reduced in MOLM-13 cells, as indicated by WB assay (Figure 2B,E, *P*<0.001).

Furthermore, following the knockdown of *UBA3*, *UBE2M* and *RBX1*, NEDD8 NEDDylation of CUL1-4 was significantly inhibited (Figure 2C,F–H, *P<*0.001). The NEDDylation of CUL5 was suppressed by the knockdown of *UBA3* but up-regulated by the knockdown of *UBE2M* and *RBX1*. Study showed that UBE2M paired RBX1 to regulate the NEDDylation of CUL1-4, while UBE2F paired RBX2 to regulate that of CUL5 in an E2-RING-dependent manner [32]. We inferred that, UBE2F was relatively active, thus promoting NEDDylation of CUL5 after UBE2M was knocked down by shRNA. In addition, the p53 signaling pathway was activated after *UBA3*, *UBE2M* and *RBX1* were knocked down, and the expression of p53 and p21 was significantly up-regulated. (Figure 2D,I–K, *P<*0.001).

### MLN4924 inhibited the proliferation and induced the apoptosis of AML cells

**Figure 3:**
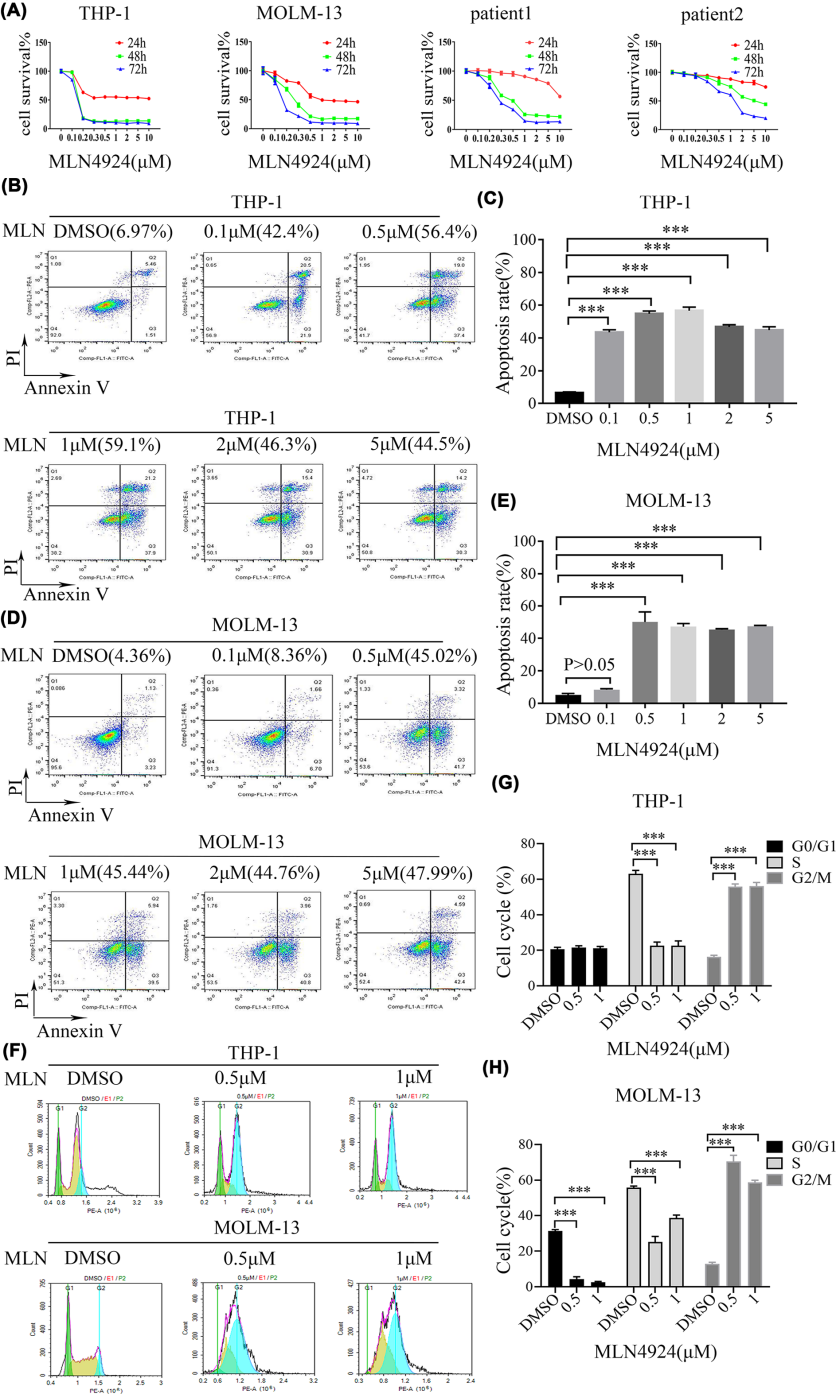
MLN4924 inhibited the proliferation and induced the apoptosis of AML cells (**A**) MLN4924 significantly inhibited the proliferation of THP-1 cells, MOLM-13 cells and the primary cells collected from two AML patients in a concentration- and time-dependent manner. (**B–E**) The numbers of apoptotic cells of THP-1 and MOLM-13 cells significantly increased following treatment with MLN4924 (except for the 0.1 μM dose group of MLN4924 in MOLM-13 cells, *P*>0.05). (**F–H**) MLN4924 induced cell cycle arrest in both THP-1 and MOLM-13 cells at the G2/M phase, and significantly increased the cells at G2/M phase; ***P<0.001. The means among multiple groups were compared by one-way ANONA and LSD-*t* test. Each experiment was carried out in triplicate.

MLN4924 has been shown to possess anti-tumor activity against several malignant tumor types [33–39]. Therefore, the effect of this compound was examined in AML cells in this work. In CCK-8 assays, MLN4924 significantly inhibited the proliferation of THP-1 and MOLM-13 cells. The proliferation of primary cells collected from two AML patients was also inhibited by MLN4924. Typically, MLN4924 at the concentration of ≥1 μM had stronger effects, with the strongest effect being obtained at 72 h (Figure 3A).

The FCM results demonstrated that the apoptotic cell frequency significantly increased following 24 h MLN4924 treatment in THP-1 cells (*P*<0.001, Figure 3B,C). Even at the dose of 0.1 μM, the apoptotic cell frequency was significantly increased (42.4% versus 6.97%, *P*<0.001). MLN4924, except for the 0.1 μM dose group of MLN4924 (*P*>0.05), also induced the apoptosis of MOLM-13 cells (Figure 3D,E, *P*<0.001). Moreover, MLN4924 induced cell cycle arrest in both THP-1 and MOLM-13 cell, as evidenced by the significantly increased proportion of cells at the G_2_/M phase (Figure 3F–H, *P*<0.001).

### MLN4924 inhibited the NEEDylation of CULs in AML cells

**Figure 4:**
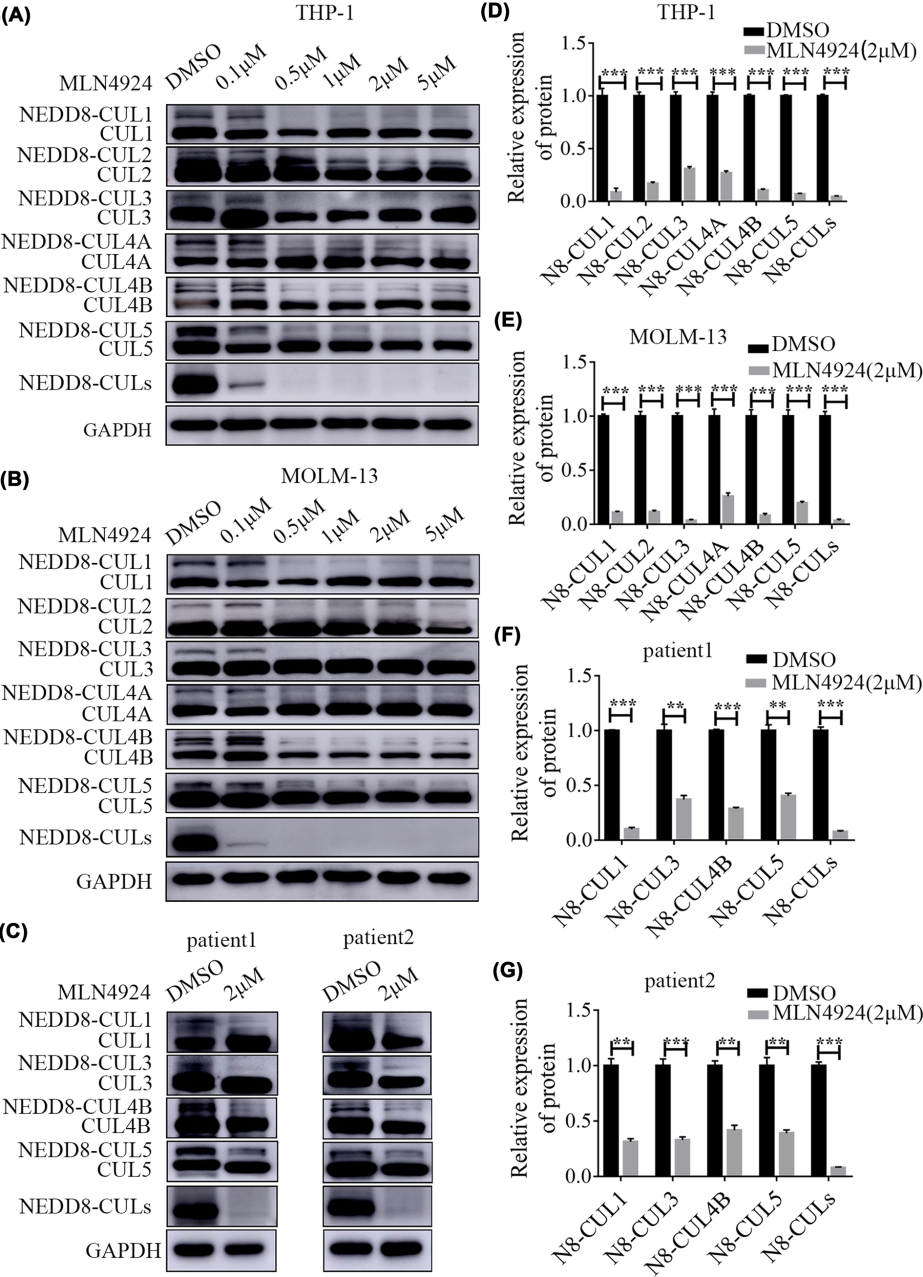
MLN4924 inhibited the NEDDylation of CULs in AML cells (**A**) MLN4924 significantly inhibited the NEDD8 NEDDylation of CUL1-5 in THP-1 cells. (**B**) In MOLM-13 cells, the NEDDylation of CUL1-5 was also inhibited. (**C**) MLN4924 inhibited the NEDD8 NEDDylation of CUL1-5 in primary AML cells collected from two AML patients. (**D–G**) Statistical analysis revealed that MLN4924 inhibited the NEDD8 NEDDylation of CULs in THP-1 cells; ***P<0.001, **P<0.01. An independent sample *t*-test was adopted to compare the means between two groups. Each experiment was conducted in trplicate.

MLN4924 is a newly discovered inhibitor of NAE [8]. MLN4924 inhibits NEDD8 binding to CULs proteins and thus decreases the NEDDylation of CULs proteins, including CUL1-5. It has been reported that CUL1, CUL2, CUL4A and CUL5 are efficiently deNEDDylated by MLN4924 in MCF breast cancer cells [4]. In the present study, MLN4924 significantly inhibited the NEDD8 NEDDylation of CUL1-5 in THP-1 cells (Figure 4A,D) and MOLM-13 cells (Figure 4B,E) treated with MLN4924 for 48 h. MLN4924 significantly reduced the NEDD8-modified CULs (*P*<0.001). Moreover, in primary cells collected from two AML patients, MLN4924 (2 μM) inhibited the NEDD8 NEDDylation of CUL1-5 proteins (Figure 4C,F,G).

### MLN4924 activated the p53 signaling pathway in AML cells

**Figure 5:**
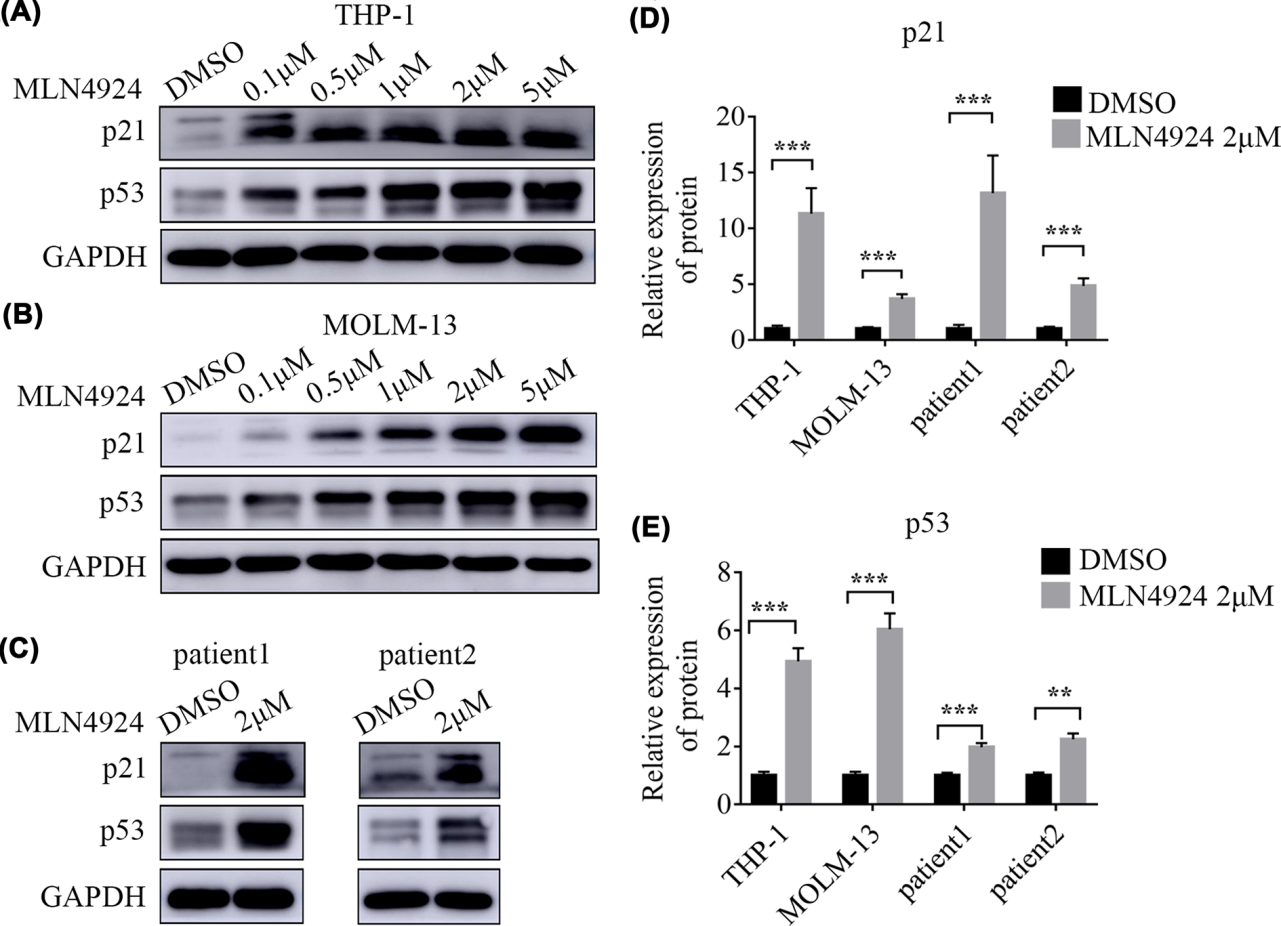
MLN4924 activated the p53 signaling pathway in AML cells (**A,B**) In THP-1 and MOLM-13 cells, the expression levels of p21 and p53 proteins were significantly up-regulated after MLN4924 treatment. (**C**) In primary AML cells harvested from two AML patients, MLN4924 increased the expression of both p21 and p53 proteins. (**D,E**) Statistical analysis indicated that the expression of p21 and p53 was up-regulated; ***P<0.001, **P<0.01. An independent sample *t*-test was conducted to compare the means between two groups. Each experiment was carried out in triplicate.

To further investigate the mechanism by which MLN4924 induced the apoptosis of AML cells, the p53 signaling pathway was examined. As revealed by WB assay, 48 h treatment with MLN4924 significantly increased the protein expression levels of p21 and p53 in THP-1 cells (Figure 5A) and MOLM-13 cells (Figure 5B). Similarly, MLN4924 had the same effects on primary cells collected from two AML patients (Figure 5C).

### MLN4924 inhibited the growth of xenograft tumors in mice

**Figure 6:**
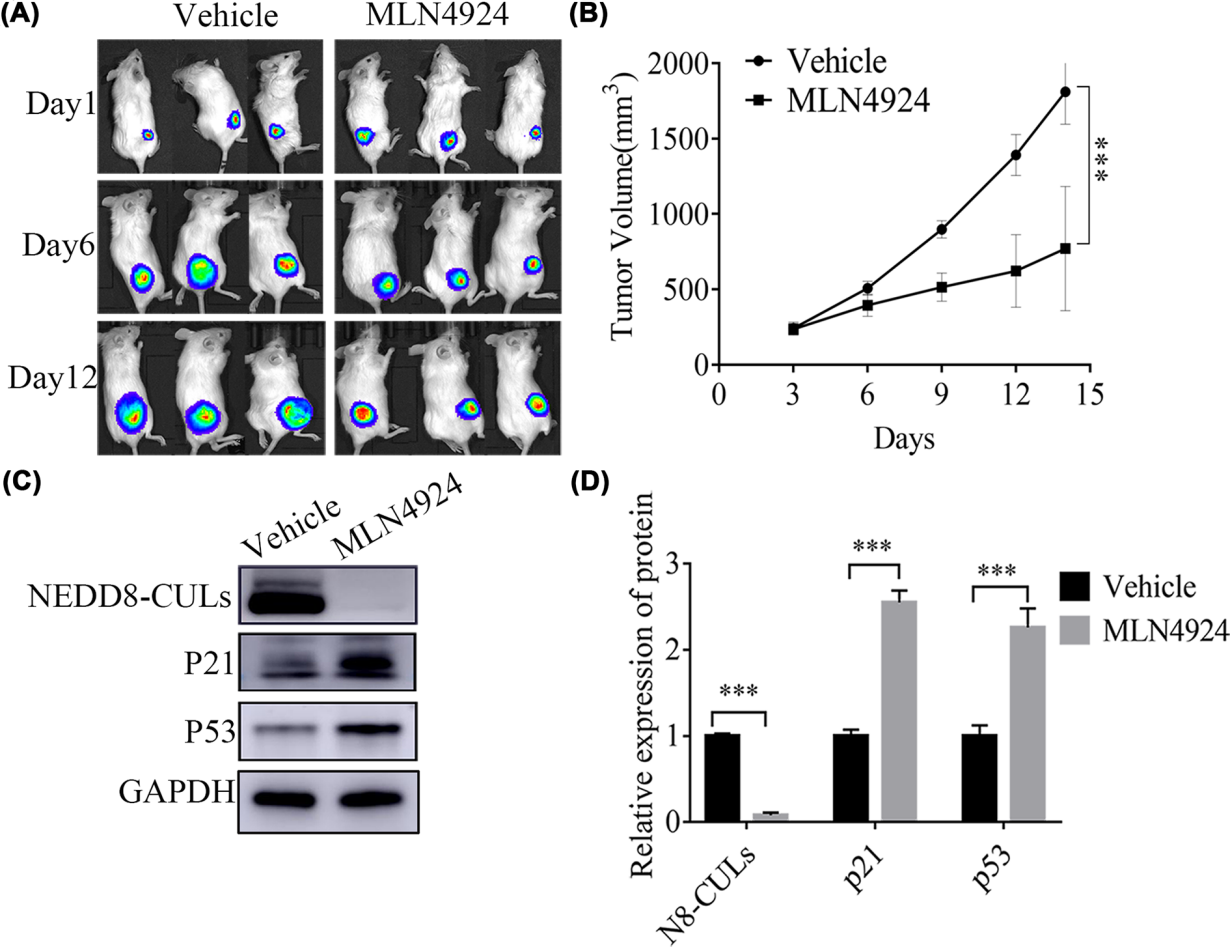
MLN4924 inhibited the growth of xenograft tumors in mice (**A**) The xenograft tumors were detected by *in vivo* bioluminescence imaging. (**B**) Compared with the control group, the growth of the xenograft tumors in the experimental group was significantly inhibited. (**C,D**) The NEDDylation of CULs was inhibited and the protein expression levels of p21 and p53 significantly increased in xenograft tumors of mice treated with MLN4924 (*n*=5). ***P<0.001. An independent sample t-test was conducted to compare the means between two groups. Each experiment was carried out in triplicate.

As demonstrated by the aforementioned *in vitro* experiments, MLN4924 inhibited the proliferation of AML cells. Therefore, the present study sought to examine this effect *in vivo* with xenograft tumors. The results suggested that MLN4924 suppressed the growth of tumors in mice. Compared with the control group, the growth of xenograft tumors in the experimental group was significantly reduced (Figure 6A,B). In addition, MLN4924 was confirmed to be safe in our experiment, with no significant toxic or side effects. WB assay suggested that the NEDDylation of CULs was inhibited, whereas the expression of p21 and p53 significantly increased, in xenograft tumors of mice that were treated with MLN4924 (Figure 6C,D, *P*<0.001), consistent with the *in vitro* experimental results.

## Discussion

The expression levels of NEDD8, E1 NEDD8-activating enzymes (NAE1/UBA3) and the NEDD8 conjugation enzyme E2 (UBE2M/UBE2F) have been shown to be up-regulated in various solid tumors, such as lung adenocarcinoma and lung squamous cell carcinoma, intrahepatic cholangiocarcinoma, liver cancer, colorectal cancer, glioblastoma, head and neck tumor, esophageal squamous cell carcinoma and renal cell carcinoma relative to adjacent normal tissues [15–22]. Similarly, the NEDD8 E3 ligases, DCN1 and RBX1/2, are also up-regulated in several types of human cancer types [25,40,41]. The up-regulation of enzymes related to NEDD8 NEDDylation is associated with disease progression and reduced patient OS [16–18,20,21]. However, to the best of our knowledge, the expression of molecules related to NEDDylation pathway in AML has not been fully examined. The present study demonstrated that the mRNA levels of *NEDD8*, *UBA3*, *UBE2M* and *RBX1* were up-regulated in AML patients, consistent with the aforementioned previous studies. Moreover, survival analysis indicated that the OS rate of patients with overexpression of these genes was lower than that in patients with low-expression levels, which was an independent prognostic factor for worse OS in AML patients. Thus, *NEDD8*, *UBA3*, *UBE2M* and *RBX1* may serve as the potential prognostic biomarkers for AML. Our short observation time might have some influence on the analysis results. In future, we will continue to follow up the patients to further analyze their survival.

UBA3, UBE2M and RBX1 are important for the proliferation and apoptosis of cancer cells. Indeed, the proliferation of M14 cells is suppressed *in vitro* and *in vivo* after UBA3 silencing with RNA interference [23]. UBE2M silencing disrupted the DNA damage response [24]. RBX1 silencing can inhibit cancer cell growth by inducing apoptosis, cell cycle arrest at G2-M phase and senescence [25]. Suppression of the NEDDylation pathway with shRNA targeting on NEDD8 and RBX1 sensitized AML cells toward azacytidine at the sublethal concentrations [26]. To further explore the functions of *UBA3*, *UBE2M* and *RBX1* in AML, these three genes were knocked down with shRNA. In MOLM-13 cells, UBA3, UBE2M and RBX1 knockdown inhibited the NEDDylation of CUL1-4. In addition, the NEDDylation of CUL5 was suppressed by the knockdown of *UBA3*, and up-regulated by the knockdown of *UBE2M* and *RBX1*. Study showed that UBE2M paired RBX1 to regulate the NEDDylation of CUL1-4, while UBE2F paired RBX2 to regulate that of CUL5 in an E2-RING-dependent manner [32]. We inferred that *UBE2F* was relatively active, thus promoting NEDDylation of CUL5 after UBE2M was knocked down by shRNA. The expression of p21 and p53 increased following the knockdown of *UBA3*, *UBE2M* and *RBX1*. In addition, the p53 signaling pathway was activated. Thus, it might be hypothesized that *UBA3*, *UBE2M* and *RBX1* silencing attenuated the function of enzymes related to the NEDDylation pathway, thereby inhibiting the NEDDylation of CULs and promoting the accumulation of their substrates, such as p21 and p53.

In the present study, the lentivirus was only transfected into MOLM-13 cells, but not THP-1 cells. In future studies, the transfection conditions and methods will be optimized to replicate the current findings in more cell lines.

MLN4924 (pevonedistat) is an inhibitor of NAE, which has been widely studied in various solid tumors [33–39,42–45]. MLN4924 inhibited NEDDylation to play an antitumor role, and induced apoptosis, senescence, autophagy, angiogenesis inhibition, inflammatory response and chemosensitization/radiosensitization [46]. In AML, MLN4924 induced DNA re-replication and damage by disrupting nucleotide metabolism and augments the efficacy of cytarabine [47]. MLN4924 also partially inhibited the transduction of mTOR signaling [48]. Swords et al. [49] found that MLN4924 induced AML cell death and led to the inhibition of NF-κB activity, DNA damage, and the generation of reactive oxygen species (ROS). MLN4924 alone or in combination with azacytidine could induce apoptosis by up-regulating NOXA in AML and synergize with Bcl-2 inhibitors [50,51]. Moreover, MLN4924 decreased the binding of NF-κB to the microRNA-155 promoter and down-regulated microRNA-155 in FLT3-ITD AML cells [52]. However, the effect of MLN4924 on p53 signaling pathway in AML has rarely been studied. In AML, p53 mutations are associated with chemoresistance and a high risk of relapse [53]. Study has found that MLN4924 activates the p53 tumor suppressor via the RPL11/RPl5-Mdm2 pathway [54]. p53 is not only modified by ubiquitination but also the substrate of NEDD8 NEDDylation. Both NEDD8 and ubiquitin modification of p53 were regulated by Mdm2, while the latter promoted the binding of ubiquitin and NEDD8 to p53 independently [55]. In colorectal cancer, p53 was identified as an important mediator of the apoptotic response to MLN4924 [33]. p21 was thought to induce tumor growth inhibition through the activity of wild-type p53 [56]. p53 can induce p21 expression in response to cellular stress, such as DNA damage or oxidative stress. In addition to cell cycle arrest, p21 also plays an important role in senescence in both p53-dependent and non-dependent manners [57,58]. In response to the activation of p53 transcription factor, p21 induction can lead to tumor growth stagnation by inhibiting the cyclin-kinase complexes, proliferative nuclear antigens, transcription factors and co-activators [57]. The MLN4924-induced cell senescence and death have been observed in various cancer cell types, which were mainly dependent on p21. For example, following MLN4924 treatment, p21 significantly accumulated, while p21 knockout significantly inhibited the senescence induced by MLN4924 [59–61]. MLN4924 is a strong inhibitor of CUL NEDDylation [12,13]. In addition, following 6 h treatment with MLN4924, CUL1, CUL2, CUL4A and CUL5 are efficiently deNEDDylated in MCF breast cancer cells [4]. In the present study, the p53 signaling pathway was activated by MLN4924 in AML cells. The expression of p53 and p21 proteins increased. Besides, the proliferation of AML cells was significantly inhibited by MLN4924. MLN4924 promoted the apoptosis of THP1 and MOLM-13 cells and induced cell cycle arrest. According to our results, MLN4924 significantly inhibited the NEDDylation of CUL1-5 in AML cells. MLN4924 formed a covalent adduct with NEDD8, which was catalyzed by the NAE. The MLN4924-NEDD8 adduct was similar to the adenosylated NEDD8 in that it could closely bind to the active site of NAE and block the activity of NAE enzyme, thus preventing the subsequent enzymatic reactions [10,11]. MLN4924 inhibited NEDD8 binding to CULs proteins and thus decreased the NEDDylation of CULs proteins, including CUL1-5. Therefore, a decrease in NEDD8-CULs was observed. It was hypothesized that MLN4924 inhibited the NEDDylation of CULs and inactivated CRLs in AML, leading to the accumulation of CRL substrates, including p21 and p53. In this way, the p53 signaling pathway was activated by MLN4924, which inhibited the proliferation of AML cells and induced their apoptosis. Thus, MLN4924 exerted an anti-leukemia effect and might represent a potential research avenue for AML treatment.

In the *in vivo* experiments, MLN4924 induced the apoptosis of vascular smooth muscle cells via p53 and p62, and improved neointimal hyperplasia by promoting the apoptosis of mouse smooth muscle via p53 [8]. A phase-1b trial on elderly patients with AML unfit for high-dose induction therapy showed that MLN4924 was well tolerated in combination with azacytidine. The overall response rate (ORR) was 50% and the median duration of remission was 8.3 months [62]. In the present study, MLN4924 had an inhibitory effect on AML xenograft tumor growth in mice. Moreover, MLN4924 was well tolerated, with no obvious side effects. In xenograft tumors, MLN4924 inhibited the NEDDylation of CULS and increased the expression of p21 and p53 proteins. Thus, MLN4924 inhibited the NEDD8 NEDDylation to inactivate CRLs, leading to the accumulation of CRL substrates and ultimately inhibiting tumor growth.

In conclusion, the present findings demonstrated that the NEDDylation pathway was dysregulated in AML. *NEDD8*, *UBA3*, *UBE2M* and *RBX1* may represent the potential prognostic biomarkers and novel therapeutic targets for AML. MLN4924 exerted an antitumor effect both *in vivo* and *in vitro* by activating the p53 signaling pathway. Therefore, inhibition of the NEDDylation pathway may represent a potential treatment for AML.

## Data Availability

The datasets used and/or analyzed during the current study are available from the corresponding author on reasonable request.

## Abbreviations

AML: acute myeloid leukemia
APPBP1: amyloid β precursor protein-binding protein 1
CCK-8: Cell Counting Kit-8
CRL: cullin-ring ligases
DMSO: dimethyl sulfoxide
ECL: enhanced chemiluminescence
FAB: French–American–British
FBS: fetal bovine serum
FCM: flow cytometry
KM: Kaplan–Meier
NAE: NEDD8-activating enzyme
NEDD8: neural precursor cell-expressed developmentally down-regulated 8
OS: overall survival
PVDF: polyvinylidene fluoride
RBX1: RING box protein-1
ROC curve: receiver operating characteristic curve
ROC1: regulator of cullin-1
RT-qPCR: real-time fluorescent quantitative polymerase chain reaction
SD: standard deviation
SDS-PAGE: sodium dodecyl sulfate polyacrylamide gel electrophoresis
shRNA: short hairpin RNA
UBA3: ubiquitin-like modifier activating enzyme 3
UBE2M: ubiquitin-conjugating enzyme 2M
WB: Western blotting
